# Sensorimotor Activity and Network Connectivity to Dynamic and Static Emotional Faces in 7-Month-Old Infants

**DOI:** 10.3390/brainsci11111396

**Published:** 2021-10-24

**Authors:** Ermanno Quadrelli, Elisa Roberti, Silvia Polver, Hermann Bulf, Chiara Turati

**Affiliations:** 1 Department of Psychology, University of Milano-Bicocca, Edificio U6, Piazza dell’Ateneo Nuovo 1, 20126 Milano, Italy; e.roberti@campus.unimib.it (E.R.); s.polver@campus.unimib.it (S.P.); hermann.bulf@unimib.it (H.B.); chiara.turati@unimib.it (C.T.); 2 NeuroMI, Milan Center for Neuroscience, 20126 Milano, Italy

**Keywords:** sensorimotor cortex, mu rhythm, functional connectivity, emotion, faces, infant

## Abstract

The present study investigated whether, as in adults, 7-month-old infants’ sensorimotor brain areas are recruited in response to the observation of emotional facial expressions. Activity of the sensorimotor cortex, as indexed by µ rhythm suppression, was recorded using electroencephalography (EEG) while infants observed neutral, angry, and happy facial expressions either in a static (*N* = 19) or dynamic (*N* = 19) condition. Graph theory analysis was used to investigate to which extent neural activity was functionally localized in specific cortical areas. Happy facial expressions elicited greater sensorimotor activation compared to angry faces in the dynamic experimental condition, while no difference was found between the three expressions in the static condition. Results also revealed that happy but not angry nor neutral expressions elicited a significant right-lateralized activation in the dynamic condition. Furthermore, dynamic emotional faces generated more efficient processing as they elicited higher global efficiency and lower networks’ diameter compared to static faces. Overall, current results suggest that, contrarily to neutral and angry faces, happy expressions elicit sensorimotor activity at 7 months and dynamic emotional faces are more efficiently processed by functional brain networks. Finally, current data provide evidence of the existence of a right-lateralized activity for the processing of happy facial expressions.

## 1. Introduction

Humans are inherently social animals. The ability to quickly recognize emotions from others’ facial expressions is foundational for successfully managing multifaceted social interactions and for an adapted social life [1]. Perception and interpretation of others’ faces play a crucial role in human communication, learning about the social and physical world, regulating our emotions, and developing relationships with others. This is especially true early in life, when infants cannot rely on language to understand others’ behaviors, but mainly observe and interpret gestures and facial expressions to grasp others’ intentions and feelings [2,3]. Neurophysiological evidence has documented the recruitment of sensorimotor brain areas in response to the expression and observation of emotional faces [4,5]. Although considerable efforts have been devoted to elucidating the neural underpinnings of the early development of emotion processing (e.g., [6,7]), little is still known about the role of sensorimotor areas in the processing of facial expressions during infancy. The current study addresses this issue by investigating 7-month-old infants’ sensorimotor response to static and dynamic facial expressions of happiness and anger and how neural networks underlying the processing of emotional expressions may be organized at this age.

A substantial number of brain imaging studies indicated the existence of a complex network of brain structures involved in the processing of facial emotional expressions in adults (see [8] for a review). This network includes cortical areas, such as the extrastriate regions of the occipital cortex, the fusiform gyrus, and the superior temporal sulcus, as well as subcortical structures, such as the amygdala and insula [9]. Given that facial expressions contain both emotional and motor components, it is not surprising that several studies showed the involvement of the premotor and parietal cortex in facial expression observation and execution [10,11]. Neurophysiological evidence for the recruitment of motor brain areas in response to emotion perception derives from the discovery of mirror neurons in monkeys’ premotor and parietal cortices [12]. They are a class of sensorimotor neurons originally studied in relation to the domain of actions and intentions [13,14]. However, their function was later associated also to social processes, such as empathy and the processing of facial emotional expressions [15,16,17]. According to some authors, the recruitment of areas known to be involved in perceptual–motor coupling mechanisms speaks in favor of the hypothesis that facial expressions are recognized via a simulation mechanism (e.g., [18]). Observing someone’s emotional expression directly generates motor and somatosensory activation in the observer as if he/she is embodying and feeling similar emotional states [4]. Studies using a variety of techniques have shown that adults recruit specific sections of the premotor, parietal, and sensory cortices during both observation and imitation of the main facial emotional expressions [4,5,19].

All these studies provide us with fundamental knowledge concerning the fully developed neural underpinnings involved in the processing of emotional facial expressions. However, from a developmental perspective, it is extremely important to understand the early emergence of the brain processes implicated in reading others’ emotional reactions and the crucial steps that lead the way to the development of adult abilities. Based on adult literature, research only recently started investigating whether the motor system might be involved in processing emotional expressions in infancy and if its activity undergoes a gradual specialization during the first years of life that might be guided by early visuomotor experiences. Indeed, to date, the vast majority of studies on the neural correlates of emotion processing in the first years of life assessed infants’ attention allocation and perceptual discrimination abilities in response to facial expressions (e.g., [20,21,22]). These studies, using electroencephalography (EEG), consistently demonstrated that heightened sensitivity to happy faces persists until 7 months, when infants’ attention allocation is more pronounced in response to happy than to negative facial expressions [20,22]. Between 7 and 12 months of age, infants’ attention starts to be preferentially attracted respectively by fearful and angry faces when these are contrasted to happy faces [23], giving rise to an attentional bias towards negative expressions. Recent research also suggests that infants are sensitive to facial dynamics, which may affect their processing of emotional expressions [24,25,26], as well as infants’ attentional biases toward emotional signals [21]. Crucially, all these studies focused on the neural correlates of attentional or perceptual processing of emotional expressions, without considering the role of sensorimotor processing across the first years of life. However, as Fransson and colleagues [27] demonstrated, the functional networks observed in the infant’s brain primarily span sensorimotor and sensory areas. Thus, such networks might scaffold more advanced processes, such as those triggered by facial expressions of emotions. Indeed, since the processing of emotional faces involves several interconnected brain regions, a network perspective can help elucidate such underlying processes [26].

Recent studies collecting surface electromyography (sEMG) data from a wide range of age groups reported that observing facial gestures or emotional expressions elicits specific muscular activation patterns (e.g., [28,29,30]). For example, it was shown that 3-year-old children exhibited increased zygomaticus major activity (i.e., the primary muscle involved in smiling) in response to happy faces, while angry faces generated an increased electromyographic activation of the frontalis muscle, which is typically involved in expressing fear [31]. Furthermore, when presented with happy, angry, and fearful facial expressions, 4-month-olds did not display selective sEMG activation of the facial muscles. On the other hand, 7-month-olds showed selective activation of the zygomaticus major and frontalis muscles respectively for happy and fearful expressions, while angry expressions did not elicit a specific response [32]. 

An additional electrophysiological measure, which has recently been used to explore the motor and sensorimotor components of emotion processing, is µ rhythm suppression or desynchronization. It is an EEG measure, which is typically recorded at central scalp locations within the alpha frequency band (i.e., 6–9 Hz in infants) [33]. Mu rhythm suppression is considered as an index of activity linked to perceptual–motor coupling mechanisms, being generated in the sensorimotor cortex during both action execution and perception (e.g., [34,35,36]). Mu suppression in response to facial emotional expressions in the first years of life has recently started to be explored. Rayson and colleagues [37] recorded thirty-month-olds’ sensorimotor activation during observation of dynamic emotional (i.e., sadness and happiness) and non-emotional facial expressions (i.e., mouth opening). They showed that µ desynchronization occurred bilaterally in central clusters during observation of non-emotional mouth opening expressions, while it was found only in the right hemisphere during observation of happy and sad facial expressions [37]. The same authors recorded similar results also in 9-month-old infants. Specifically, they found significant µ desynchronization in response to the observation of happy, sad, and mouth opening facial expressions compared to scrambled faces over the right hemisphere [38]. Nonetheless, these results do not clarify whether sensorimotor activation at 9 months is specifically elicited by emotional expressions or if it is determined by the observation of faces or face movements in general. Indeed, empirical evidence indicates that, among negative emotions, recognition of sadness is characterized by a longer developmental trajectory compared to the recognition of fear and anger [39]. Thus, the fact that a similar response has been observed for a highly familiar emotional expression (i.e., happiness), a non-emotional expression (i.e., mouth opening), and an emotional expression (i.e., sadness) that is known to be recognized only later in development, seems to question the possibility that sensorimotor activation is specifically elicited by facial expressions of emotions in infancy. 

Furthermore, several neuroimaging studies (see [8]) demonstrated that the processing of facial emotional expressions is not limited to specific brain regions but requires the involvement of several interconnected brain areas (e.g., amygdala, frontal, parietal, and occipital cortices). In order to better characterize both functional and anatomical interactions between brain regions, graph theory has been proposed as an optimal way to describe brain networks and their interactions, reducing them to an abstract set of nodes and connections [40]. Within this framework, graph measures provide theoretical justification regarding what can be considered the optimal performance in an optimally organized network [41]. In a connectivity approach, brain functions are considered to emerge from synchronized activity (i.e., edges) of several information processing nodes (i.e., vertices). In a graph, the backbone structure behind brain-specific behavior, such as the perception of emotional stimuli, can be evaluated by the minimum spanning tree (MST). MST is an unbiased method to represent the essential features of brain networks, as it allows for mapping the strongest connections avoiding loops [42,43,44]. It results in a backbone graph that is thought to reflect the major qualitative properties of connectivity while allowing a better comparison across different conditions [43]. Previous reports have shown that brain networks measured through MSTs become progressively more connected with age, mirroring myelination processes and providing evidence for the usefulness of trees as a network reduction technique [41].

In terms of tree topology, two extreme shapes have been described, the first being a line in which all nodes are connected to two other nodes. The other extreme is a star in which there is a central node to which all other nodes are connected with one link [43,44]. A measure employed to characterize tree shapes is the diameter: the diameter of a tree is the longest distance (in edges) between any two nodes of a tree. The smaller the diameter, the more star-like and efficient the configuration is [43]. It has been suggested that an optimal network performance is driven by efficient communication between all vertices, requiring a smaller diameter and thus a star-like topology [42]. However, in a star-like tree the central node might easily be overloaded; therefore, the second criterion to ensure network efficiency is the prevention from hubs’ overloading by setting a maximal betweenness centrality for any tree node [42]. Consequently, the optimal tree should reflect the best possible balance between both criteria (i.e., diameter and betweenness centrality). Finally, another way to directly assess efficiency is to refer to integration measures that estimate the ease with which brain regions communicate, with shorter paths implying stronger potential for integration [45]. Within this framework, the average inverse shortest path length is a measure known as global efficiency. At higher global efficiency values, networks are more integrated and characterized by efficient communication routing [45]. 

Based on these premises, in the current study sensorimotor activation was explored by recording µ rhythm desynchronization in 7-month-olds in response to the observation of static and dynamic stimuli depicting neutral faces, as well as happy and angry emotional facial expressions. Existing behavioral and ERP findings suggest that 7-month-old infants display a heightened sensitivity to happy compared to angry expressions, possibly resulting from a greater exposure to positive expressions throughout the first months of life [23]. Thus, by presenting this age group with happy and angry facial expressions we aimed at examining whether the enhanced attentional and perceptual processing of happy over angry faces might affect sensorimotor activation. Should we observe a differential pattern of activation determined by the emotional valence of the stimuli, with greater activation generated by one of the three facial expressions in the dynamic but not in the static condition, the hypothesis of a specific sensorimotor activation for the distinct facial expressions, possibly molded by acquired sensorimotor experience, would be corroborated. More specifically, under the hypothesis that familiarity or acquired experience with emotional expressions play a role in shaping sensorimotor activity, greater activation is expected in response to happy (i.e., more familiar and experienced) compared to angry faces (i.e., less familiar and experienced) at 7 months of age. On the contrary, observation of a significant µ rhythm desynchronization when facial expressions are dynamic but not static, would indicate that sensorimotor areas are activated in response to any facial movement. Moreover, in light of results from network-based measures in developmental samples [26,46], probing brain functional organization might provide insights into the roles played by emotional and sensorimotor components in the perception of static and dynamic facial emotional expressions early in life. Specifically, we sought to explore EEG networks’ efficiency in the µ rhythm frequency band in response to dynamic and static emotional facial expressions to verify whether network organization differs between static and dynamic emotions in 7-month-old infants.

## 2. Materials and Methods

### 2.1. Participants

Thirty-eight 7-month-old infants (20 males, M age = 217 days, SD = 13 days, range = 201–233 days) were included in the final sample. Infants were randomly assigned to one of two experimental conditions so that 19 infants were presented with the dynamic condition, and 19 infants were presented with the static condition. All infants were recruited from a mixed urban environment including the metropolitan and suburban areas of Milano (Italy), were born full-term (37–42 weeks gestation), with normal birth weight (>2500 g), did not suffer of any neurological or other medical conditions, and had normal vision and hearing for their age. Twenty-two additional infants were tested but excluded from analysis due to fussiness (*N* = 8), excessive artifacts (*N* = 12), or technical problems with data collection (*N* = 2). The sample size and proportion of excluded infants are comparable to other EEG studies investigating µ rhythm with infants this age (e.g., [47,48]). Furthermore, on the basis of an a priori power analysis, an overall sample size of 28 participants was estimated to provide 80% statistical power to achieve a medium effect size (*f* = 0.25). The procedure followed ethical standards (the Declaration of Helsinki, BMJ 1991; 302:1194), and the ethical committee of the University of Milano-Bicocca approved the study protocol. Families with infants were invited in-writing based on birth records of the city of Milano and neighboring towns. The study was explained to the parents/caregivers who gave their signed informed consent. This is a secondary analysis of data from a study conducted to investigate 7-month-olds’ event-related potentials in response to static and dynamic neutral, happy and angry facial expressions (see [21] for a detailed description).

### 2.2. Stimuli

**Figure 1:**
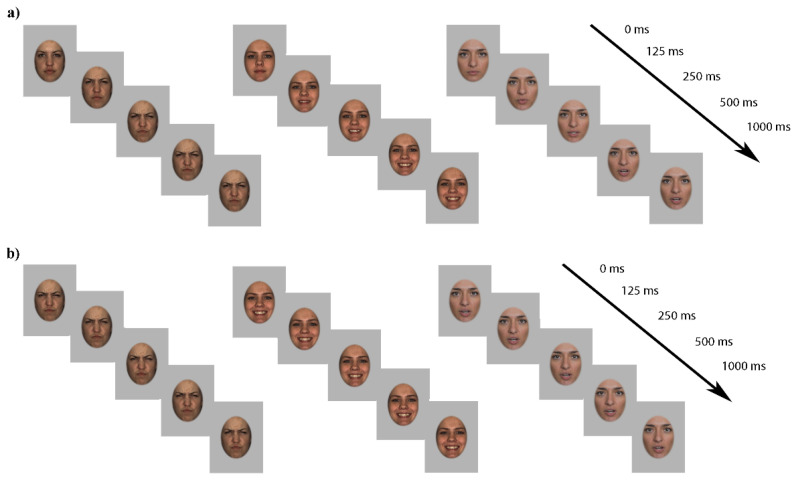
Examples of frames from videos used in the dynamic (**a**) and static (**b**) conditions representing the angry (**left**), happy (**central**) and neutral (**right**) expressions. In the static condition, the same picture depicting the full emotional expression remained on screen.

Stimuli in the dynamic condition consisted of short 1000 ms color videos of 6 female Caucasian actresses posing neutral, angry, and happy facial expressions while facing forward. Videos depicting happy and angry facial expressions were taken from the Binghampton University 4D Facial Expression database (BU-4DFE) [49]. Each video depicting an emotional face took 500 ms to reach the full expression (i.e., neutral to 100% intensity), which remained on the screen until the end of the video (i.e., for another 500 ms) (Figure 1). Videos illustrating neutral expressions were recorded at our laboratory and represented three actresses posing a neutral face and then moving their mouths without producing any sound. In the static condition, all stimuli consisted of photographs depicting the full emotional expression (100%) extracted from videos used in the dynamic condition and presented for 1000 ms. Different identities were used for each emotion so that there was no overlap between the identities posing the three facial expressions. All stimuli were cropped into an oval shape using the software Adobe Photoshop. This was done to remove hair and external features and facilitate the processing of featural and configural cues indicative of each emotion [50,51]. Indeed, it is known that the external facial features largely captivate infants’ attention [50], and that covering the hair promotes the processing of the internal portion of the face (e.g., [52]). All faces subtended 15.3° and 10.5° of visual angle vertically and horizontally when viewed from approximately 60 cm and were displayed against a grey background. As also reported in Quadrelli and colleagues [21], nineteen adult raters (13 females) screened and selected the stimuli for their emotional valence by completing a survey in which they had to identify each emotion by selecting from the list of the six basic emotional expressions. In the static condition, neutral, happy, and angry expressions were correctly identified by 100%, 86%, and 76% of the raters, respectively, while in the dynamic condition, they were correctly identified by 97%, 91%, and 97% of the raters, respectively. Raters were also requested to assign to the face a score ranging from −10 (i.e., angry) to 10 (i.e., happy) to describe the intensity of the expressed emotion, with 0 corresponding to the absence of emotional expression. Wilcoxon signed-ranks tests performed for each emotion on the intensity scores indicated that both happy (Dynamic: M = 7.16, SD = 0.84; Static: M = 7.20, SD = 0.81), Z > 3.83; *p*s < 0.001, $\eta_{p}^{2}$ > 1.63, and angry expressions (Dynamic: M = −6.89, SD = 1.31; Static: M = −6.56, SD = 1.08), Z > −3.84; *p*s < 0.001, $\eta_{p}^{2}$ > 1.63, were perceived as equally different from neutral expressions, which instead were properly perceived as non-emotional (Dynamic: M = 0.42, SD = 0.89; Static: M = 0.42, SD = 0.89), Z > 1.81; *p*s > 0.07, $\eta_{p}^{2}$ > 0.38. All stimuli were also equalized for luminance, which did not differ between emotional expressions both in the dynamic and static) conditions (Kruskal–Wallis H test χ^2^(2) > 5.60; *p*s > 0.08; $\eta_{p}^{2}$ > 0.60). Furthermore, to control for possible differences in terms of quantity of movement between emotional categories, an analysis of the motion content of the stimuli was performed through an established procedure [53,54]. Specifically, the amount of movement for each video within each emotion was evaluated by computing the variation of luminance between pairs of contiguous frames. These luminance variation estimates were then averaged within each emotional expression in order to verify the presence of a difference in movement between facial expressions. The comparison between the overall amount of motion displayed in the videos depicting the three dynamic facial expressions did not reveal any difference in the amount of motion between neutral, happy, and angry expressions (Kruskal–Wallis H test χ^2^(2) > 3.60; *p*s > 0.16; $\eta_{p}^{2}$ > 0.28). 

### 2.3. Procedure

Participants sat on their mother’s lap, in a behavioral state of quiet alertness, at approximately 60 cm from a 24-inch monitor in a dimly lit, audiometric and electrically shielded cabin. Stimuli were presented using E-Prime software v2.0 (Psychology Software Tools Inc., Pittsburgh, PA, USA). Mothers were instructed to sit as still as possible and remain silent throughout the experimental session to prevent any acoustic interference. An infrared camera, hidden over the monitor and feeding into the data acquisition computer outside the testing cabin, allowed the experimenters to record the whole procedure. The data acquisition computer streamed the live video of the infants’ faces and bodies to enable the experimenter to pause or interrupt the session in case the infant became too fussy. All six face identities, either in a dynamic or static condition, were presented in a random order to each infant, with the only constraint that models expressing the same emotion could not be displayed more than three times consecutively. The experimental session ended when infants attended to the maximum number of trials (*N* = 180) or got tired of the experiment. A trial consisted of a 1000 ms stimulus presentation followed by an interstimulus interval that varied randomly between 900 and 1100 ms. The experimenter had the possibility to present a looming fixation point between trials to redirect the infant’s attention to the monitor. The caregivers were taught to keep their child attention to the screen ahead without distracting them by pointing or vocalizing.

### 2.4. EEG Recording and Processing

Continuous EEG was recorded using a 128-electrode Geodesic Sensor Net connected to a NetAmps 300 amplifier (Electrical Geodesic In., Eugene, OR, USA). Data were sampled at 500 and recorded with respect to the vertex electrode. The signal was amplified with a bandpass filter of 0.1 to 100 Hz, and impedances were checked before the beginning of each session and deemed adequate if lower than 50 KΩ. and re-referenced to the average reference. Pre-processing of the signal was performed using Netstation v4.6.4. Data were further high-pass filtered offline (0.3 Hz) and segmented to 2400 ms segments, comprising 1000 ms before and 1400 ms after stimulus onset. Automated artifact detection was applied to segmented data to reject segments and/or specific electrodes in which the signal exceeded ±200 µV. The results of the automatic procedure were subject to further visual inspection based on the video recorded throughout the experiment. This was done to ensure that any trial in which the infant did not attend to the screen or made any gross or fine limb or head movements was subsequently excluded and to confirm the presence of eye movements, eye-blinks, and any other body movement previously detected by the automated algorithm. Trials were excluded if more than 15% of channels in a trial were marked as bad. Subsequently, of the accepted trials, individual bad channels were replaced by an automated algorithm using spherical spline interpolation. Only infants with a minimum of 5 artifact-free trials per condition were included in the final analyses [55]. The mean number of artifact-free trials contributing to analyses was 7.33 (happiness: 7.53, SD = 2.12; anger: 7.26, SD = 2.74; neutral: 7.21, SD = 2.53) in the dynamic condition, and 7.98 (happiness: 8.26, SD = 2.46; anger: 7.58, SD = 2.20; neutral: 8.11, SD = 1.41) in the static condition. No significant differences were found between conditions and emotions in the number of artifact-free trials (all *p*s > 0.07). 

### 2.5. Time–Frequency Analysis

Time–frequency analysis was performed using WTools (see [56]) on each artifact-free trial applying a continuous wavelet transform with Morelet wavelets at 1 Hz intervals in the 3 to 20 Hz range. After similar studies investigating µ rhythm desynchronization (e.g., [34,36,57]) or performing time–frequency analysis to explore other oscillatory responses in infancy (e.g., [56,58]), the absolute value (i.e., the amplitude, not the power) of the resulting complex coefficients was estimated. The first and the last 400 ms of each epoch were removed to eliminate distortion introduced by the wavelet transform, and a 500 ms baseline period beginning 600 ms before stimulus onset was selected. As in previous work demonstrating that in infants of this age, the 6–9 Hz frequency band is most sensitive to movement [33,59], we averaged activity over this range. The averaged activation in the 6–9 Hz range of the 500 ms baseline was then subtracted from averaged activation recorded after stimulus onset. We calculated the average wavelet coefficients within infants by taking the mean across trials. Likewise, existing studies examining sensorimotor activity in response to emotional expressions in infancy [37,38], activation recorded over a cluster of electrodes disposed over the left- (30, 31, 36, 37, 41, 42, 53, and 54) and the right-hemispheres (79, 80, 86, 87, 93, 103, 104, and 105) was analyzed. The scalp locations of these left- and right-central electrode clusters correspond to the locations of C3 and C4 in the international 10–20 electrode placement system. Statistical analyses were conducted on the average activity in the 6–9 Hz range extracted from these two regions in the 400–800 ms time window. This time window was chosen based on visual inspection of the data, indicating that activation elicited by the employed emotional expressions reached its peak across participants within this time window. All individual averages were also visually inspected to make sure that the chosen time window was appropriate. In addition, because we wanted to know whether sensorimotor suppression while infants observed the emotional expressions was specific to the central region or extended to the occipital region [60], we also analyzed activity recorded from a cluster of channels over the occipital cortex (70, 71, 75, 76, 83), corresponding to O1/Oz/O2 according to the international 10–20 electrode placement system. All statistical tests were conducted on a 0.05 level of significance (two-tailed). When the ANOVAs yielded significant effects, pairwise comparisons including ≤3 means were performed by applying t-tests and the Fisher’s least significant difference procedure [61], and Holm–Bonferroni correction were used where appropriate [62].

### 2.6. Network Analysis

Following the pre-processing pipeline in Netstation, data were passed to FieldTrip [63] for adjacency matrices computation. To avoid issues caused by volume conduction, we computed the scalp current source density (CSD) using the second-order derivative (the surface Laplacian) of the EEG potential distribution through a spherical spline method. On CSD data, the debiased weighted phase lag index (DWPLI) [64] was computed as a connectivity measure. The frequency range of interest was the 6–9 Hz µ range. One weighted adjacency matrix based on DWPLI values was derived per subject, per condition, and per emotion. To avoid issues caused by the arbitrary choice of thresholds to remove spurious connections, graph metrics were computed referring to minimum-spanning-tree (MST) topologies. MSTs calculation overcomes the bias of network density and degree without any additional normalization step [43,44]. In fact, it is mathematically defined as the subnetwork of the original weighted network that connects all nodes in the network without forming loops and has the minimum total weight of all possible spanning trees [44,46]. The fixed number of edges confers advantages to MST analyses when evaluating network efficiency [65]. In the present study, MSTs were constructed based on the weighted networks with Kruskal’s algorithm [66]. After MSTs construction, link weights were binarized.

In order to measure network efficiency, we referred to integration measures, as they provide a reliable way to characterize the brain’s capacity to rapidly combine specialized information from distributed brain regions. Measures of integration illustrate this concept by estimating the ease with which brain regions communicate and are commonly based on the concept of a path [45,67]. Paths are sequences of distinct nodes and links, and lengths of paths estimate the potential for functional integration between brain regions, with shorter paths implying a stronger potential for integration [67]. To measure integration, we computed the average inverse shortest path length, a measure known as global efficiency [68]. In addition, to characterize MSTs shapes and all the possible configurations in between a star and a line, we computed the diameter [43]. Both the global efficiency and the diameter were computed by converting MST matrices to corresponding distance matrices. Finally, to assess networks’ overloading, we referred to the betweenness centrality. The betweenness centrality is the fraction of all shortest paths in the network that contain a given node. Nodes with high values of betweenness centrality participate in a large number of shortest paths. As the name implies, we can think of this measure as indexing the extent to which a node lies “between” other pairs of nodes. If information travels through a network along the shortest path, then nodes that lie on many shortest paths will mediate a high proportion of traffic and thus represent central elements of the network. In this sense, such a node might play a controlling role in the passage of information through the network or act as a traffic bottleneck [67]. Here we considered the maximum betweenness centrality value per connectivity matrix normalized to the 0–1 range (BCnorm) to assess the overall centrality configuration of connectivity matrices. The betweenness centrality was computed on length matrices, obtained by mapping connections weights to lengths in the original adjacency matrices in order to assign the smallest values to the shortest distances (i.e., the strongest connections) of one node to each other node in the network. As such, length matrices can be considered the inverse of connectivity matrices and allow the subsequent MST analysis to provide a robust estimation of a highly connected and efficient subnetwork [65]. For MSTs and metrics computation, we used the Brain Connectivity Toolbox [45] and custom MATLAB functions.

## 3. Results

### 3.1. Time–Frequency

To compare the scalp distribution µ rhythm desynchronization over central electrode clusters during the observation of dynamic and static happy, angry, and neutral facial expressions in 7-month-old infants, we employed a 2 × 2 × 3 repeated measures analysis of variance (ANOVA) with experimental condition (dynamic, static) as between-subject factor, and electrode cluster (C3, C4) and emotion (happiness, anger, neutral) as within-subject factors.

**Figure 2:**
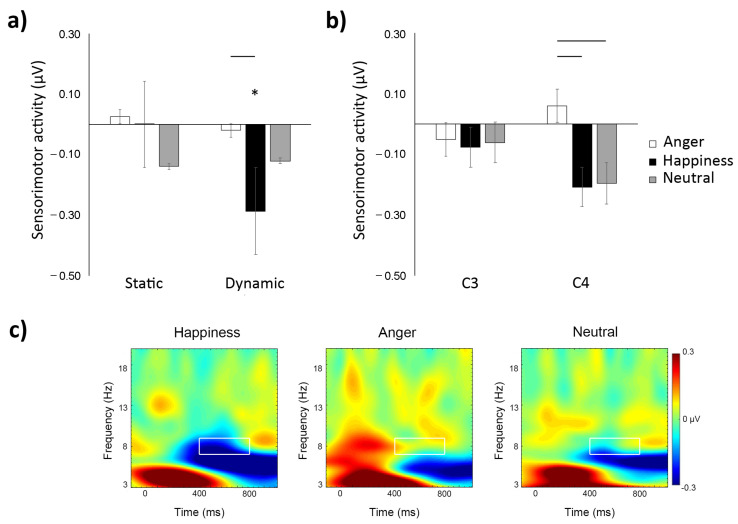
Illustrations of time–frequency results. The upper panels (**a**,**b**) display mean alpha activity over the selected electrode clusters covering sensorimotor areas during the observation of static and dynamic neutral, happy, and angry facial expressions. Significant suppression from baseline and significant comparisons between conditions are illustrated, * *p* < 0.05. Error bars represent the standard errors of the means. In the bottom panel (**c**), time–frequency plots display baseline-corrected activity respectively for dynamic happy, angry, and neutral faces over sensorimotor areas (i.e., C3 and C4).

The ANOVA yielded a significant main effect of emotion, *F*(2,72) = 3.57; *p* = 0.03, $\eta_{p}^{2}$ = 0.09, with happy expressions (M = −0.14 µV; SD = 0.35 µV) eliciting greater sensorimotor alpha suppression compared to angry faces (M = 0.004 µV; SD = 0.35 µV), irrespectively of the experimental condition (all other *p*s > 0.05). However, the main effect was qualified by a significant emotion by electrode cluster interaction, *F*(2,72)= 4.35; *p* = 0.02, $\eta_{p}^{2}$ = 0.11. Post hoc comparisons showed that there was less sensorimotor alpha suppression for angry expressions (M = 0.06 µV; SD = 0.39 µV) compared to happy (M = −0.21 µV; SD = 0.35 µV, *t* (37) = 4.55; *p* < 0.001, d = 0.66) and neutral faces (M = −0.19 µV; SD = 0.36 µV, *t* (37) = 3.20; *p* = 0.04, *d* = 0.20) over C4 (i.e., right hemisphere) (Figure 2b). All other comparisons did not attain statistical significance (all *p*s > 0.57). Furthermore, a significant emotion by experimental condition interaction, *F*(2,72) = 3.51; *p* = 0.03, $\eta_{p}^{2}$ = 0.09, was also found. Post hoc comparisons were conducted separately for each experimental condition. The analysis of the dynamic experimental condition revealed that happy facial expressions (M = −0.29 µV; SD = 0.29 µV) elicited more sensorimotor alpha suppression compared to angry faces (M = −0.02 µV; SD = 0.39 µV), *t* (18) = −4.11; *p* < 0.001, *d* = 0.94 (Figure 2a). No other comparison attained statistical significance (all *p*s > 0.09). Conversely, the analysis of the static experimental condition did not reveal any statistically significant difference between activities elicited by any of the facial expressions (all *p*s > 0.07).

Additionally, to examine the magnitude of sensorimotor alpha suppression as compared to baseline in both experimental conditions and both electrode clusters in response to neutral, happy, and angry facial expressions, one-sample t-tests were performed. In the dynamic experimental condition, sensorimotor alpha suppression in response to happy expressions over the C4 electrode cluster (M = −0.38 µV; SD = 0.27 µV) was significantly different from zero, *t* (18) = −6.23; *p* < 0.01, *d* = 1.43. No other comparisons attained significance in the dynamic condition (all *p*s > 0.15). In the static experimental condition, activity for all facial expressions over the two electrode clusters failed to attain statistical significance (all *p*s > 0.17).

Finally, to ascertain whether µ rhythm desynchronization was specific to central sites, similarly to previous studies (e.g., [55,69,70,71]) we performed a separate repeated-measures ANOVA with emotion (happiness, anger, neutral) as within-subject factor and experimental condition (dynamic, static) as between-subject factor on activation over occipital electrodes. The analysis performed on the occipital cluster (O1/Oz/O2) did not yield any significant main or interaction effect (all *p*s > 0.10). However, activation of occipital regions during the observation of emotional expressions in both experimental conditions was significantly different from baseline activation (static neutral: M = −1.00 µV, SD = 0.98 µV; static happiness: M = −1.21 µV, SD = 1.20 µV; static anger: M = −0.97 µV, SD = 1.04 µV; dynamic neutral: M = −1.05 µV, SD = 1.14 µV; dynamic happiness: M = −1.52 µV, SD = 1.27 µV; dynamic anger: M = −1.21 µV, SD = 1.10 µV; all *p*s < 0.001). Thus, while there was a modulation of sensorimotor suppression at central channels in response to the observed emotional expressions and the specific experimental conditions, all facial expressions in both experimental conditions elicited a significant activation as compared to baseline over the occipital cluster.

### 3.2. Network Analysis

In order to compare the efficiency of EEG activations in the specific µ rhythm frequency band in response to dynamic and static emotional facial expressions, we computed a 2 × 2 repeated-measures ANOVA, with experimental condition (dynamic, static) as between-subject factor and emotion (happiness, anger, neutral) as within-subject factor, for each graph property (i.e., global efficiency, diameter, and BCnorm). All statistical tests were conducted on a 0.05 level of significance (two-tailed) and corrected with the false discovery rate (FDR) [72]. 

**Figure 3:**
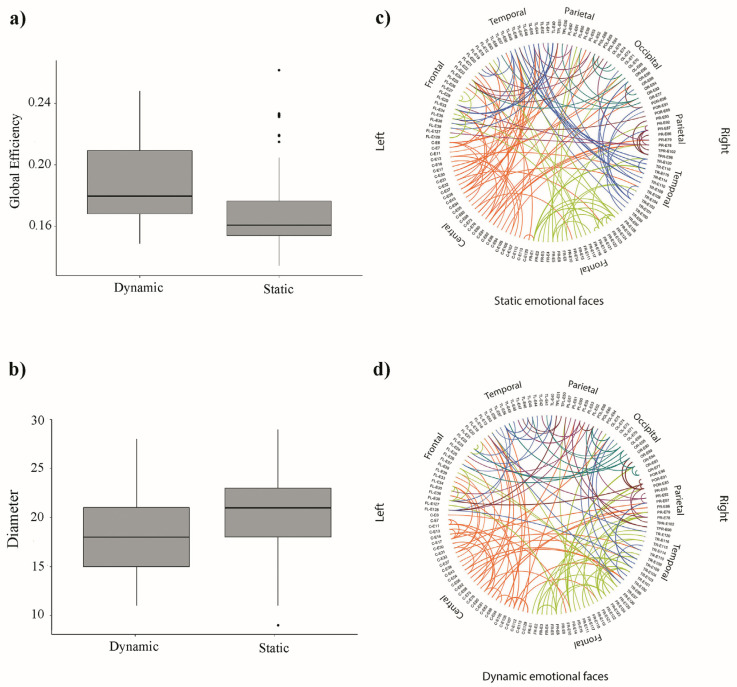
Illustrations of the functional connectivity results. The left panels show box plots depicting the difference in global efficiency (**a**) and diameter (**b**) between the dynamic and static conditions. The right panels depict circular graphs representing the topological organization of MSTs in the static (**c**) and dynamic (**d**) conditions.

Results showed a significant main effect of experimental condition on global efficiency values, *F*(1,108) = 10.14, *p* < 0.01, $\eta_{p}^{2}$ = 0.086. Post hoc comparisons showed that the dynamic condition (M = 0.19, SD = 0.02) entailed higher global efficiency compared to the static condition (M = 0.17, SD = 0.02), *t* (108) = 3.18, *p* < 0.01, *d* = 0.6 (Figure 3a). No other main or interaction effect attained statistical significance (all *p*s > 0.37). Regarding diameter values, results highlighted a significant main effect of experimental condition, *F*(1,108) = 5.54, *p* < 0.05, $\eta_{p}^{2}$ = 0.049. Post hoc comparisons showed that the dynamic condition (M = 18.24, SD = 4.16) entailed lower diameter compared to the static condition (M = 20.19, SD = 4.69), *t* (108) = −2.35, *p* < 0.05, d = −0.43 (Figure 3b). No other main or interaction effect attained statistical significance (all *p*s > 0.13). No significant results were found for BCnorm (all *p*s > 0.2) indexing no network overloads.

## 4. Discussion

Studying the neural bases of the development of emotion perception can provide useful insights into the mechanisms by which the ability to interact with the social world develops. For this reason, the present study investigated whether the observation of faces expressing different emotions in a dynamic compared to a static fashion was able to generate activation of the sensorimotor cortex. In particular, we sought to verify if neutral, angry, and happy expressions were capable of eliciting sensorimotor activation in 7-month-old infants. Our results provide evidence of differential modulation of µ rhythm desynchronization in response to static and dynamic facial expressions at 7-months of age. Indeed, happy facial expressions elicited greater sensorimotor activation compared to angry faces in the dynamic experimental condition, while no difference was found between facial expressions in the static condition, when sensorimotor activity did not also differ from baseline. This finding is in line with considerable evidence suggesting that dynamic information is beneficial for various aspects of face processing across the lifespan. Several studies demonstrated that dynamic facial expressions enhanced emotion recognition abilities [73,74] and generated stronger emotion-specific mimicry responses in adults [75,76]. Research demonstrated that 5-month-olds presented with dynamic facial expressions display an attentional bias towards fearful faces at an earlier age [77,78], and that 7-month-old infants showed a differential modulation of event-related potential responses to dynamic vs. static emotional faces [21]. Adding to this body of evidence, the current results further suggest that the perception of dynamic compared to static emotional faces augments sensorimotor activation to happy compared to angry faces. Dynamic facial expressions are more similar to those we encounter in everyday life, and they constitute a powerful means for emotional communication compared to static expressions. 

The differential activation pattern elicited by happy and angry faces in the dynamic condition further extends evidence of sensorimotor sensitivity to emotional expressions in infancy. It is possible to hypothesize that sensorimotor areas might be more sensitive to dynamic happy faces compared to angry faces in the first months of life. Indeed, several mechanisms could underlie this activation pattern and lead to a facilitation in sensorimotor activation for positive emotions, ranging from infants’ spontaneous preference for happy facial expressions [79], greater familiarity with the perceptual configuration of dynamic happy faces, and greater daily experience in interactions involving happy faces [80,81] However, it is to note that verbal interactions of parents with their infants in the first months of life can be considered a pervasive experience. Thus, it can be affirmed that the silently talking faces included in the dynamic neutral condition are as familiar as a happy expression. Notwithstanding, only dynamic happy expressions (and not angry and neutral faces) elicited a significant activation when compared against the baseline, suggesting that the effects we observed in the current study are not due to the familiarity of the stimuli per se, but to the familiarity of a face expressing a positive emotion.

Notably, activation elicited by happy faces over the right hemisphere in the dynamic experimental condition was the only case in which µ rhythm suppression attained significance as compared to baseline. This right hemisphere dominance is consistent with evidence from existing studies on sensorimotor activation to emotional expressions in toddlerhood [37] and adults [82]. Indeed, this activation pattern extends evidence highlighting the prominent role of the right hemisphere in the processing of emotional information from faces [83]. However, differently from previous studies with older infants and toddlers [37,38], our 7-month-olds did not show significant activation in response to negative expressions over the right hemisphere. The absence of activation in response to angry faces, together with the specific response to happy expressions, might suggest that perceptual and motor experience with smiling in the first months of life could lead to the maturation of a specialized sensorimotor activity for processing emotional expressions [2]. Interestingly, it appears from current results that the lateralization of the neural response to faces with positive emotional value does not imply a prolonged developmental trajectory. Rather, these data support the idea that the dominance of the right hemisphere for processing happy expressions is present very early in life. According to the neuroconstructivist perspective, an active and observational experience would be responsible for the gradual specialization of perceptual-motor couplings [84] and, as outlined by Leppanen and Nelson [2], infants’ early experience with faces might lead to a rapid attunement of face-sensitive cortical structures to the more experienced facial expressions.

The lack of significant sensorimotor activation in response to angry faces in the dynamic condition is in line with previous investigations exploring spontaneous facial reactions to angry emotional expressions in infancy and early childhood [31,32]. Likely, this result is at least partially due to the insufficiently developed ability of 7-month-old infants to extract the emotional value of angry facial expressions. Moreover, differences in exposure to angry compared to happy expressions in the first months of life may be responsible for a longer time course in the emergence of sensorimotor activation in response to angry facial expressions. Indeed, it is only by the end of the first year of life, when infants begin to actively explore the environment through locomotion, that caregivers report an increase in their expression of anger toward their siblings. In this vein, it can be hypothesized that the limited exposure to negative facial expressions within the first months of life might influence and inhibit sensorimotor activation in response to angry facial expressions.

Irrespective of the experimental condition, significant µ rhythm suppression was identified in response to happy and neutral faces compared to angry expressions over the right central electrode cluster. The reasons for the finding that neutral expressions elicit greater sensorimotor activity compared to angry faces are unclear. In general, the processing of neutral expressions has been scarcely investigated in developmental studies. The current result, together with the lack of difference between happy and neutral faces, could support the hypothesis that infants perceived and interpreted the neutral expressions, in the static as well as in the dynamic condition, as positively connoted. Indeed, existing literature suggests that when neutral expressions are employed, infants regulate their behavioral responses in a similar fashion to infants exposed to positive expressions [85]. Likewise, since our neutral videos represented silent talking faces, it is possible that 7-month-old infants perceived these stimuli as highly salient. Indeed, existing data suggest that by between 4 and 8 months of age, infants shift their attention from the eyes to the mouth when observing someone talking [86], and are capable of discriminating between languages just by watching videos of silent visual speech [87]. Thus, as also indicated by Rayson and colleagues [37,38], infants’ sensorimotor areas may be sensitive to such communicative cues and tend to resonate with such perceived social behavior.

Differently from results obtained specifically for dynamic faces, static facial expressions did not elicit a significant modulation compared to baseline and did not show a differential sensorimotor activation pattern in 7-month-old infants. Results from previous studies employing static stimuli demonstrated that observation of happy faces generated greater µ rhythm suppression compared to expressions of disgust in adults [82], and that 7-month-olds exhibited greater zygomaticus activation in response to the observation of happy compared to angry static expressions [28]. The lack of neural differentiation between happy and angry static faces in our study might be due to methodological aspects. It is possible that infants in our study were not able to fully encode happy and angry facial expressions in the static condition since the stimuli were presented very briefly (i.e., 1 s) compared to the static faces employed in the previous sEMG study (i.e., 5 s) [28]. Thus, it is plausible that the absence of dynamic and more ecologically valid information together with the shorter stimulus duration may have exerted a detrimental impact on the observed activation pattern in the static condition.

Importantly, no differences were highlighted across conditions and emotional expressions over the occipital cluster. Conversely, as previously noted, µ rhythm desynchronization was modulated differently both as a function of the dynamic vs. static experimental condition and the emotional expressions over the central electrode clusters. No significant differences in occipital alpha activity emerged between emotional expressions. However, our results also highlight the presence of significant alpha activity in all conditions and emotions as compared to baseline. These findings are consistent with previous reports showing that in infants [69] and adults [88], µ rhythm suppression over central regions is accompanied by alpha desynchronization recorded from occipital electrode clusters. Occipital alpha is known to be linked to visual attention [89,90]. In this vein, the occipital alpha attenuation we have found regardless of the observed condition and emotional expression might reflect the involvement of an attentional component during the observation of salient stimuli, such as static or dynamic facial expressions. 

Regarding MSTs results, we observed an increased global efficiency and decreased diameter in response to dynamic stimuli compared to static ones. As noted for time–frequency results, we highlighted a differential µ rhythm response to dynamic stimuli, entailing a more efficient and organized processing of naturalistic stimuli and differential processing of static compared to dynamic stimuli already at 7-months of age. This observation is consistent with reports suggesting a prominent role of 6 to 9 Hz oscillations for inducing optimal network configurations during development [46]. More generally, it is known that network structure balance is progressively optimized with remarkable reorganization throughout development, shifting from a more random to a well-organized configuration [91]. Connectivity increases across functional networks, leading to a more functionally distributed and hierarchical pattern [92,93]. Coherently, the development of brain regions dedicated to high-level cognitive processing is accompanied by increasing efficiency in the coordination of different functional networks and by increased integration of unimodal information [93]. As such, the absence of processing differences between neutral, happy, and angry faces might be explained by referencing a still not-well organized brain organization. In fact, as indexed by time–frequency results, infants are sensitive to facial dynamics, which affect their processing of emotional expressions. Thus, it is possible that as the infant’s brain matures, a rough preference for dynamic stimuli, as indexed by increased efficiency, might turn into a more fine-tuned specialization for emotional processing. Such specialization, even if not yet strongly embedded in the brain’s organization, appears to be already underway at 7 months as indexed by increased sensorimotor activation over the right hemisphere elicited in response to happy dynamic facial expressions. Finally, it is important to note that we did not observe network overloads across experimental conditions, indexing that, beyond their differences, both static and dynamic emotional faces are easily processed by infants. These results provide further confirmation of the central role of face perception for social world exploration in infants [21].

## 5. Conclusions

Taken together, results from the current study provide evidence of a modulation of sensorimotor activity and of a more selective or tuned activation of networks elicited in response to dynamic emotional expressions as early as 7 months of age. Additionally, our findings speak in favor of the existence of a right-lateralized sensorimotor activation in response to dynamic expressions of happiness, consistent with the hypothesis of an early specialized right-hemisphere dominance for the processing of more experienced happy facial expressions.

## Data Availability

The data that support the findings of this study will be made available upon request. Requests may be sent to the corresponding author (ermanno.quadrelli@unimib.it).

## References

[1] Adolphs R. (2002). Recognizing emotion from facial expressions: Psychological and neurological mechanisms. Behav. Cogn. Neurosci. Rev..

[2] Leppänen J.M., Nelson C.A. (2008). Tuning the developing brain to social signals of emotions. Nat. Rev. Neurosci..

[3] Leppänen J.M. (2011). Neural and developmental bases of the ability to recognize social signals of emotions. Emot. Rev..

[4] Carr L., Iacoboni M., Dubeau M.-C., Mazziotta J.C., Lenzi G.L. (2003). Neural mechanisms of empathy in humans: A relay from neural systems for imitation to limbic areas. Proc. Natl. Acad. Sci. USA.

[5] Leslie K.R., Johnson-Frey S.H., Grafton S.T. (2004). Functional imaging of face and hand imitation: Towards a motor theory of empathy. NeuroImage.

[6] Crespo-Llado M.M., Vanderwert R., Roberti E., Geangu E. (2018). Eight-month-old infants’ behavioral responses to peers’ emotions as related to the asymmetric frontal cortex activity. Sci. Rep..

[7] Jessen S., Grossmann T. (2015). Neural signatures of conscious and unconscious emotional face processing in human infants. Cortex.

[8] Haxby J.V., Hoffman E.A., Gobbini M. (2000). The distributed human neural system for face perception. Trends Cogn. Sci..

[9] Eimer M., Holmes A. (2007). Event-related brain potential correlates of emotional face processing. Neuropsychologia.

[10] Dapretto M., Davies M.S., Pfeifer J.H., Scott A.A., Sigman M., Bookheimer S., Iacoboni M. (2005). Understanding emotions in others: Mirror neuron dysfunction in children with autism spectrum disorders. Nat. Neurosci..

[11] Van Der Gaag C., Minderaa R.B., Keysers C. (2007). Facial expressions: What the mirror neuron system can and cannot tell us. Soc. Neurosci..

[12] di Pellegrino G., Fadiga L., Fogassi L., Gallese V., Rizzolatti G. (1992). Understanding motor events: A neurophysiological study. Exp. Brain Res..

[13] Fadiga L., Craighero L. (2004). Electrophysiology of action representation. J. Clin. Neurophysiol..

[14] Rizzolatti G., Fadiga L., Gallese V., Fogassi L. (1996). Premotor cortex and the recognition of motor actions. Cogn. Brain Res..

[15] Enticott P.G., Johnston P.J., Herring S., Hoy K., Fitzgerald P.B. (2008). Mirror neuron activation is associated with facial emotion processing. Neuropsychologia.

[16] Gallese V. (2003). The roots of empathy: The shared manifold hypothesis and the neural basis of intersubjectivity. Psychopathology.

[17] Kaplan J.T., Iacoboni M. (2006). Getting a grip on other minds: Mirror neurons, intention understanding, and cognitive empathy. Soc. Neurosci..

[18] Bastiaansen J., Thioux M., Keysers C. (2009). Evidence for mirror systems in emotions. Philos. Trans. R. Soc. B Biol. Sci..

[19] Pohl A., Anders S., Schulte-Rüther M., Mathiak K., Kircher T. (2013). Positive facial affect—An fMRI study on the involvement of insula and amygdala. PLoS ONE.

[20] Peltola M.J., Leppänen J.M., Mäki S., Hietanen J.K. (2009). Emergence of enhanced attention to fearful faces between 5 and 7 months of age. Soc. Cogn. Affect. Neurosci..

[21] Quadrelli E., Conte S., Cassia V.M., Turati C. (2019). Emotion in motion: Facial dynamics affect infants’ neural processing of emotions. Dev. Psychobiol..

[22] Taylor-Colls S., Pasco Fearon R.M. (2015). The effects of parental behavior on infants’ neural processing of emotion expressions. Child. Dev..

[23] Grossmann T., Striano T., Friederici A.D. (2007). Developmental changes in infants’ processing of happy and angry facial expressions: A neurobehavioral study. Brain Cogn..

[24] Addabbo M., Longhi E., Marchis I.C., Tagliabue P., Turati C. (2018). Dynamic facial expressions of emotions are discriminated at birth. PLoS ONE.

[25] Missana M., Grigutsch M., Grossmann T. (2014). Developmental and individual differences in the neural processing of dynamic expressions of pain and anger. PLoS ONE.

[26] Rotem-Kohavi N., Oberlander T., Virji-Babul N. (2017). Infants and adults have similar regional functional brain organization for the perception of emotions. Neurosci. Lett..

[27] Fransson P., Åden U., Blennow M., Lagercrantz H. (2011). The functional architecture of the infant brain as revealed by resting-state fMRI. Cereb. Cortex.

[28] Datyner A., Henry J.D., Richmond J.L. (2017). Rapid facial reactions in response to happy and angry expressions in 7-month-old infants. Dev. Psychobiol..

[29] De Klerk C.C., Bulgarelli C., Hamilton A., Southgate V. (2019). Selective facial mimicry of native over foreign speakers in preverbal infants. J. Exp. Child. Psychol..

[30] Hashiya K., Meng X., Uto Y., Tajiri K. (2018). Overt congruent facial reaction to dynamic emotional expressions in 9–10-month-old infants. Infant Behav. Dev..

[31] Geangu E., Quadrelli E., Conte S., Croci E., Turati C. (2016). Three-year-olds’ rapid facial electromyographic responses to emotional facial expressions and body postures. J. Exp. Child. Psychol..

[32] Kaiser J., Crespo-Llado M.M., Turati C., Geangu E. (2017). The development of spontaneous facial responses to others’ emotions in infancy: An EMG study. Sci. Rep..

[33] Marshall P.J., Bar-Haim Y., Fox A.N. (2002). Development of the EEG from 5 months to 4 years of age. Clin. Neurophysiol..

[34] De Klerk C.C., Johnson M., Heyes C.M., Southgate V. (2014). Baby steps: Investigating the development of perceptual-motor couplings in infancy. Dev. Sci..

[35] Fox N.A., Bakermans-Kranenburg M.J., Yoo K.H., Bowman L.C., Cannon E.N., Vanderwert R.E., Ferrari P.F., van Ijzendoorn M. (2016). Assessing human mirror activity with EEG mu rhythm: A meta-analysis. Psychol. Bull..

[36] Quadrelli E., Geangu E., Turati C. (2019). Human action sounds elicit sensorimotor activation early in life. Cortex.

[37] Rayson H., Bonaiuto J.J., Ferrari P.F., Murray L. (2016). Mu desynchronization during observation and execution of facial expressions in 30-month-old children. Dev. Cogn. Neurosci..

[38] Rayson H., Bonaiuto J., Ferrari P.F., Murray L. (2017). Early maternal mirroring predicts infant motor system activation during facial expression observation. Sci. Rep..

[39] Izard C.E. (2007). Basic emotions, natural kinds, emotion schemas, and a new paradigm. Perspect. Psychol. Sci..

[40] Bullmore E., Barnes A., Bassett D.S., Fornito A., Kitzbichler M., Meunier D., Suckling J. (2009). Generic aspects of complexity in brain imaging data and other biological systems. NeuroImage.

[41] Smit D.J., de Geus E.J., Boersma M., Boomsma D.I., Stam C.J. (2016). Life-span development of brain network integration assessed with phase lag index connectivity and minimum spanning tree graphs. Brain Connect..

[42] Boersma M., Smit D.J., Boomsma D.I., de Geus E., De Waal H.A.D.-V., Stam C.J. (2013). Growing trees in child brains: Graph theoretical analysis of electroencephalography-derived minimum spanning tree in 5- and 7-year-old children reflects brain maturation. Brain Connect..

[43] Stam C., Tewarie P., van Dellen E., van Straaten E., Hillebrand A., Van Mieghem P. (2014). The trees and the forest: Characterization of complex brain networks with minimum spanning trees. Int. J. Psychophysiol..

[44] Tewarie P., van Dellen E., Hillebrand A., Stam C. (2015). The minimum spanning tree: An unbiased method for brain network analysis. NeuroImage.

[45] Rubinov M., Sporns O. (2010). Complex network measures of brain connectivity: Uses and interpretations. NeuroImage.

[46] Tóth B., Urbán G., Háden G.P., Márk M., Török M., Stam C.J., Winkler I. (2017). Large-scale network organization of EEG functional connectivity in newborn infants. Hum. Brain Mapp..

[47] Gerson S.A., Bekkering H., Hunnius S. (2015). Short-term motor training, but not observational training, alters neurocognitive mechanisms of action processing in infancy. J. Cogn. Neurosci..

[48] Paulus M., Hunnius S., van Elk M., Bekkering H. (2012). How learning to shake a rattle affects 8-month-old infants’ perception of the rattle’s sound: Electrophysiological evidence for action-effect binding in infancy. Dev. Cogn. Neurosci..

[49] Yin L., Chen X., Sun Y., Worm T., Reale M. A high-resolution 3D dynamic facial expression database. Proceedings of the 8th International Conference on Automatic Face and Gesture Recognition.

[50] Leitzke B.T., Pollak S.D. (2016). Developmental changes in the primacy of facial cues for emotion recognition. Dev. Psychol..

[51] Richoz A.-R., Lao J., Pascalis O., Caldara R. (2018). Tracking the recognition of static and dynamic facial expressions of emotion across the life span. J. Vis..

[52] Mondloch C.J., Geldart S., Maurer D., Le Grand R. (2003). Developmental changes in face processing skills. J. Exp. Child. Psychol..

[53] Grossmann T., Jessen S. (2017). When in infancy does the “fear bias” develop?. J. Exp. Child. Psychol..

[54] Pichon S., de Gelder B., Grèzes J. (2009). Two different faces of threat. Comparing the neural systems for recognizing fear and anger in dynamic body expressions. NeuroImage.

[55] Cannon E.N., Yoo K.H., Vanderwert R., Ferrari P.F., Woodward A., Fox N.A. (2014). Action experience, more than observation, influences mu rhythm desynchronization. PLoS ONE.

[56] Parise E., Csibra G. (2013). Neural responses to multimodal ostensive signals in 5-month-old infants. PLoS ONE.

[57] Pomiechowska B., Csibra G. (2017). Motor activation during action perception depends on action interpretation. Neuropsychologia.

[58] Csibra G., Davis G., Spratling M.W., Johnson M.H. (2000). Gamma oscillations and object processing in the infant brain. Science.

[59] Marshall P.J., Meltzoff A. (2011). Neural mirroring systems: Exploring the EEG mu rhythm in human infancy. Dev. Cogn. Neurosci..

[60] Cuevas K., Cannon E.N., Yoo K., Fox N.A. (2014). The infant EEG mu rhythm: Methodological considerations and best practices. Dev. Rev..

[61] Gudgeon A.C., Howell D.C. (1994). Statistical methods for psychology. J. R. Stat. Soc. Ser. D. Stat..

[62] Abdi H. (2010). Holm’s sequential Bonferroni procedure. Encycl. Res. Des..

[63] Oostenveld R., Fries P., Maris E., Schoffelen J.-M. (2010). FieldTrip: Open source software for advanced analysis of MEG, EEG, and invasive electrophysiological data. Comput. Intell. Neurosci..

[64] Vinck M., Oostenveld R., van Wingerden M., Battaglia F., Pennartz C.M.A. (2011). An improved index of phase-synchronization for electrophysiological data in the presence of volume-conduction, noise and sample-size bias. NeuroImage.

[65] Tillem S., Van Dongen J., Brazil I., Baskin-Sommers A. (2018). Psychopathic traits are differentially associated with efficiency of neural communication. Psychophysiology.

[66] Kruskal J.B. (1956). On the shortest spanning subtree of a graph and the traveling salesman problem. Proc. Am. Math. Soc..

[67] Fornito A., Zalesky A., Bullmore E. (2016). Fundamentals of Brain Network Analysis.

[68] Latora V., Marchiori M. (2001). Efficient behavior of small-world networks. Phys. Rev. Lett..

[69] Filippi C.A., Cannon E.N., Fox N.A., Thorpe S., Ferrari P.F., Woodward A. (2016). Motor system activation predicts goal imitation in 7-month-old infants. Psychol. Sci..

[70] Southgate V., Vernetti A. (2014). Belief-based action prediction in preverbal infants. Cognition.

[71] Upshaw M.B., Bernier R.A., Sommerville J.A. (2016). Infants’ grip strength predicts mu rhythm attenuation during observation of lifting actions with weighted blocks. Dev. Sci..

[72] Benjamini Y., Hochberg Y. (1995). Controlling the false discovery rate: A practical and powerful approach to multiple testing. J. R. Stat. Soc. Ser. B.

[73] Ambadar Z., Schooler J.W., Cohn J.F. (2005). Deciphering the enigmatic face: The importance of facial dynamics in interpreting subtle facial expressions. Psychol. Sci..

[74] Krumhuber E., Kappas A., Manstead A. (2013). Effects of dynamic aspects of facial expressions: A review. Emot. Rev..

[75] Rymarczyk K., Biele C., Grabowska A., Majczyński H. (2011). EMG activity in response to static and dynamic facial expressions. Int. J. Psychophysiol..

[76] Weyers P., Muhlberger A., Hefele C., Pauli P. (2006). Electromyographic responses to static and dynamic avatar emotional facial expressions. Psychophysiology.

[77] Heck A., Hock A., White H., Jubran R., Bhatt R.S. (2016). The development of attention to dynamic facial emotions. J. Exp. Child. Psychol..

[78] Heck A., Hock A., White H., Jubran R., Bhatt R.S. (2017). Further evidence of early development of attention to dynamic facial emotions: Reply to Grossmann and Jessen. J. Exp. Child. Psychol..

[79] Farroni T., Menon E., Rigato S., Johnson M.H. (2007). The perception of facial expressions in newborns. Eur. J. Dev. Psychol..

[80] Hoehl S. (2013). Emotion processing in infancy. The Impact of Immigration on Children’s Development.

[81] Vaish A., Grossmann T., Woodward A. (2008). Not all emotions are created equal: The negativity bias in social-emotional development. Psychol. Bull..

[82] Moore A., Gorodnitsky I., Pineda J. (2012). EEG mu component responses to viewing emotional faces. Behav. Brain Res..

[83] Calvo M.G., Beltrán D. (2014). Brain lateralization of holistic versus analytic processing of emotional facial expressions. NeuroImage.

[84] Quadrelli E., Turati C. (2015). Origins and development of mirroring mechanisms: A neuroconstructivist framework. Br. J. Dev. Psychol..

[85] Repacholi B.M., Meltzoff A.N., Olsen B. (2008). Infants’ understanding of the link between visual perception and emotion: “If she can’t see me doing it, she won’t get angry”. Dev. Psychol..

[86] Lewkowicz D.J., Hansen-Tift A.M. (2012). Infants deploy selective attention to the mouth of a talking face when learning speech. Proc. Natl. Acad. Sci. USA.

[87] Weikum W.M., Vouloumanos A., Navarra J., Soto-Faraco S., Sebastián-Gallés N., Werker J.F. (2007). Visual language discrimination in infancy. Science.

[88] Marshall P.J., Bouquet C.A., Shipley T.F., Young T. (2009). Effects of brief imitative experience on EEG desynchronization during action observation. Neuropsychologia.

[89] Warreyn P., Ruysschaert L., Wiersema J.R., Handl A., Pattyn G., Roeyers H. (2013). Infants’ mu suppression during the observation of real and mimicked goal-directed actions. Dev. Sci..

[90] Debnath R., Salo V., Buzzell G.A., Yoo K.H., Fox N.A. (2019). Mu rhythm desynchronization is specific to action execution and observation: Evidence from time-frequency and connectivity analysis. NeuroImage.

[91] Cao M., Huang H., He Y. (2017). Developmental connectomics from infancy through early childhood. Trends Neurosci..

[92] Boersma M., Smit D.J., de Bie H.M., Van Baal G.C.M., Boomsma D.I., de Geus E.J., de Waal H.A.D.-V., Stam C.J. (2011). Network analysis of resting state EEG in the developing young brain: Structure comes with maturation. Hum. Brain Mapp..

[93] Wen X., Zhang H., Li G., Liu M., Yin W., Lin W., Zhang J., Shen D. (2019). First-year development of modules and hubs in infant brain functional networks. NeuroImage.

